# Socioeconomic factors and effect of evidence-based patient information about primary prevention of type 2 diabetes mellitus - are there interactions?

**DOI:** 10.1186/1756-0500-7-541

**Published:** 2014-08-18

**Authors:** Jutta Genz, Burkhard Haastert, Hardy Müller, Frank Verheyen, Dennis Cole, Wolfgang Rathmann, Bettina Nowotny, Michael Roden, Guido Giani, Christian Ohmann, Andrea Icks

**Affiliations:** Institute of Biometrics and Epidemiology, German Diabetes Center, Leibniz Institute at the Heinrich Heine University Düsseldorf, Auf’m Hennekamp 65, 40225 Düsseldorf, Germany; German Center for Diabetes Research (DZD), Neuherberg, Germany; mediStatistica, Lambertusweg 1b, 58809 Neuenrade, Germany; WINEG - TK - Scientific Institute for Benefit and Efficiency in Health Care, Bramfelder Straße 140, 22305 Hamburg, Germany; Institute for Clinical Diabetology, German Diabetes Center, Leibniz Institute at the Heinrich Heine University, Düsseldorf, Auf’m Hennekamp 65, 40225 Düsseldorf, Germany; Department of Endocrinology and Diabetology, University Clinics Düsseldorf, Moorenstr. 5, 40225 Düsseldorf, Germany; Heinrich Heine University Düsseldorf, Coordination Centre for Clinical Trials (KKS), Moorenstr. 5, 40225 Düsseldorf, Germany; Faculty of Medicine, Department of Public Health, Heinrich Heine University Düsseldorf, Moorenstr. 5, 40225 Düsseldorf, Germany

**Keywords:** Evidence-based patient information, Prevention of Type 2 diabetes, Socioeconomic status

## Abstract

**Background:**

Having shown in a recent randomized controlled trial that evidence-based patient information (EBPI) significantly increased knowledge on primary prevention of diabetes compared to standard patient information, we now investigated interaction between socioeconomic status (SES) and the effect of an EBPI.

**Findings:**

1,120 visitors (aged 40–70 years, without known diabetes) to the “Techniker Krankenkasse” and the “German Diabetes Center” websites were randomized. The intervention group received a newly developed on-line EBPI, the control group standard on-line information. The primary outcome measure was knowledge, classified as “good/average/poor”. We analyzed associations of knowledge with socioeconomic variables (education, vocational training, employment, subjective social status) combined with intervention effect including interactions, adjusted for possible confounding by knowledge before intervention, self–reported blood glucose measurements, blood pressure, blood lipid levels, age and gender. Logistic regression models were fitted to the subpopulation (n = 647) with complete values in these variables.

Education (high vs. low) was significantly associated with knowledge (good vs. average/poor); however, there was no significant interaction between education and intervention. After adjustment, the other socioeconomic variables were not significantly associated with knowledge.

**Conclusions:**

Socioeconomic variables did not significantly change the effect of the intervention. There was a tendency towards a lower effect where lower educated individuals were concerned. Possibly the power was too low to detect interaction effects. Larger studies using SES-specific designs are needed to clarify the effect of SES. We suggest considering the socioeconomic status when evaluating a decision aid, e.g. an EBPI, to ensure its effectiveness not only in higher socioeconomic groups.

**Trial registration:**

Current Controlled Trials ISRCTN22060616 (Date assigned: 12 September 2008).

## Findings

A way of defining the concept of health literacy is “the capacity to obtain, process and understand basic health information and services needed to make appropriate health decisions”. This is essential in the attempt to increase people’s control over their health [1]. Poor health-related knowledge limits health literacy [2]. In this context, the aim of decision aids [3] to provide evidence-based information about health conditions and their associated options, benefits, harm, probabilities and scientific uncertainties becomes important. A lower socioeconomic status of persons - not only of those with diabetes - as measured by educational status, employment and/or occupation is linked to worse health literacy, which in turn associates with poorer health and higher risk of mortality [2, 4]. Few decision aids, including those providing evidence-based patient information (EBPI), have been evaluated regarding their effectiveness among people from a range of socioeconomic backgrounds [5, 6], and results are conflicting. In a recent randomized controlled trial (registered as ISRCTN22060616), we estimated the EBPI effects on specific knowledge and components of informed patient decision-making. We found that readers of evidence-based information have significantly better knowledge of blood glucose testing issues and primary prevention of diabetes compared to those who read standard patient information [7]. This study now investigates additionally the associations between socioeconomic variables and their interactions with the effects of EBPI, using a subpopulation with the same data. Our hypothesis is that there are interactions between the intervention effect of the EBPI and the socioeconomic position.

### Study population

In cooperation with the statutory health insurance company “Techniker Krankenkasse” (TK) and the German Diabetes Center we invited visitors (without known diabetes and aged between 40 and 70 years) to the respective websites to take part in our study. For advertising purposes we posted lottery incentives on the TK web site and placed an advertisement in the TK customer magazine. Immediately after finishing the first questionnaire, a total of 1,120 individuals were randomly allocated by a stratified block randomization to either the intervention group or the control group. The intervention group received a newly developed on-line EBPI, in German, about elevated blood glucose levels and metabolic screening, derived from the best-available published research to date. The EBPI gives details about the best available evidence on the natural course of the disease, sensitivities and specificities of screening routine, and the options of primary prevention of diabetes mellitus and its effectiveness, without providing any advice. The control group was exposed to standard information from the Internet, comprising information and advice about prevention, early detection, sequelae and therapy of Type 2 diabetes mellitus in the form of a widely used online brochure and two articles from popular web sites [7]. In comparison with standard information, EBPI is more comprehensive, i.e. it communicates all treatment options together with their effectiveness, lack of effectiveness, insufficient knowledge on effectiveness, and also adverse effects. Effect estimates are provided with confidence intervals to inform about uncertainty. Its development follows the accepted steps of EBPI development [8]: 1) systematic literature research, 2/3) selection and assessment of relevant publications, 4) presentation of the main results using risk communication techniques, 5) pilot phase.

The primary outcome measure was knowledge on blood glucose testing issues and primary prevention of diabetes, classified in “good/average/poor”. It has been assessed using an eight-item multiple-choice-scale that we developed for our own use, as there was no German version of a validated instrument available.

Participation required obtaining informed consents from all participants via the Internet, in accordance with the Declaration of Helsinki [9]. The Ethics Committee of the University of Düsseldorf approved the design of the study on February 28, 2008 and the amended protocol on August 07, 2009 (Reference number: 3020). There were 334 participants who dropped out before beginning or completing the knowledge questionnaire (flow chart in [7]). A complete case analysis was performed, excluding a further 139 participants with missing values in the socioeconomic variables and with confounders of interest, resulting in a final study population of n = 647. An additional sensitivity analysis was performed including participants with missing values in confounders.

### Variables and data analysis

We included the variables knowledge (good/average and poor = 3 categories, recoded to 2), intervention (yes/no = control), the socioeconomic variables education (high: 12 or 13 years, medium: 10 years, low: 9 years or no high-school education or other kind of basic education), vocational training (yes/no), employment (yes/no) and subjective social status (ordinal, 3 values). Further variables included were knowledge before intervention (ordinal, 4 questions = 0 to 4 correct answers = 4 values), self-reported former blood glucose measurements (surveyed: yes/no), increased blood pressure (high: yes/no) and blood lipid levels (elevated: yes/no), age and gender as potential confounders [7, 10]. Demographic and socioeconomic variables of the study population were described by frequency tables and mean ± standard deviation depending on the distribution of the variables, stratified by the intervention and control groups. Logistic regression models with dependent variable “knowledge” (2 categories: good vs. average and poor) were fitted to the data. Bivariate models, each considering an intervention along with one other variable as covariates, were fitted, and also a further model using all variables. Intervention, gender, age and all significant predictor variables were included in a final model. In this final model, interaction variables between each socioeconomic variable and intervention were included additionally, and some stratified models with socioeconomic variables were calculated. In a sensitivity analysis, we calculated complete continuation logit regression models [11], according to the study protocol [10]. Furthermore, a sensitivity analysis was performed by including missing indicators for predictors in the final model instead of excluding participants with missing values. All analyses were performed using the Statistical Analysis Systems SAS (SAS for Windows, Release 9.3 SAS Institute Inc. Cary, NC, USA). All statistical tests were two-sided with a level of significance of 5%, if not stated otherwise.

## Results

**Figure 1:**
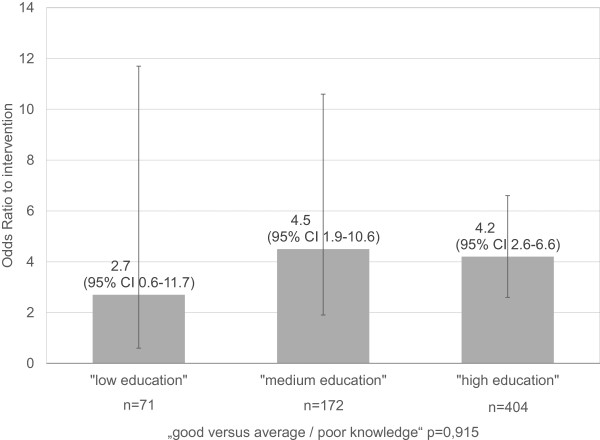
**Interaction between education and intervention; Odds Ratios adjusted for age, gender, blood lipid levels and knowledge before intervention; stratified models.**

From a dropout analysis (647 included vs. 473 excluded participants) significant differences were concluded in age (50 ± 7 vs. 51 ± 8 years), vocational training (98 vs. 94%), employment (80% vs. 70%) and “poor knowledge before intervention” (36 vs. 45%). Dropout was not significantly associated with intervention, gender, education and subjective social status. Table 1 presents characteristics of the included study participants. Regarding the results of the bivariate logistic models and the big model including all covariates, besides the intervention, only education and employment among the socioeconomic variables have shown significant associations (ORs [95% CI] of education high/low 2.9 [1.5-5.8], vocational training 0.5 [0.2-1.7], employment 1.7 [1.1-2.7], subjective social ranking high/low 0.7 [0.4-1.2]). After adjustment for confounders, only education remained (adjusted OR for employment 1.1 [0.6-2.0]). In the final model the covariates intervention, education, age, gender, blood lipid level and “knowledge before the intervention” were included. Intervention and education (high vs. low) were significantly associated with knowledge (good vs. average/poor) (OR 4.0; 95% CI 2.7 to 6.0 and OR 2.3; 95% CI 1.1 to 4.7, respectively). Stratified regression models showed that in the category “low education”, knowledge was about three times higher in the intervention group than in the control group; however, the confidence intervals were broad. In the category “medium education” knowledge was 4.5 and in the category “high education” about four times higher (Figure 1). The differences (ratios) between the odds ratios in the strata correspond to the interaction effect between education and intervention on the overall population, which was rated as non-significant (p = 0.915). The continuation logit analysis showed comparable results (data not shown). Furthermore, in a sensitivity analysis, which included 774 of all 786 participants with non-missing outcome, the results were similar (data not shown).

**Table 1:** **Participant characteristics (n = 647)**

	Intervention group		Control group	
	n = 313		n = 334	
Variable	%	N	%	N
Gender (male)	39.6	124	38.3	128
Age 50–70 years	43.8	137	44.3	148
	50.1 ± 7.4		50.0 ± 7.6	
Education level: high (12 or 13 years)	59.7	187	65.0	217
Education level: medium (10 years)	27.8	87	25.5	85
Education level: low (9 years or no high-school education or other kind of basic education)	12.5	39	9.6	32
Vocational training: yes	97.4	305	98.2	328
Employment: yes	79.9	250	79.9	267
Subjective social status *(3 values):* high	16.6	52	16.2	54
Subjective social status *(3 values):* medium	54.6	171	51.2	171
Subjective social status *(3 values):* low	28.8	90	32.6	109
Good knowledge (outcome)				
Education level: high (12 or 13 years)	49.2	92/187	19.4	42/217
Education level: medium (10 years)	31.0	21/87	10.6	9/85
Education level: low (9 years or no high-school education or other kind of basic education)	23.1	9/39	9.4	3/32

## Discussion

We found a hint that EBPI may be less effective when applied to individuals with low education compared to those with average or high education. However, there was no significant impact of the socioeconomic variables education, vocational training, employment or by the subjective social status on the EBPI effect on knowledge. A systematic review about complex interventions to improve the health of people with limited literacy [1] found that several interventions have been less suitable in individuals with lower literacy or education. However, results are conflicting: Trevena et al. [6] and Smith et al. [5] evaluated a decision aid for colorectal cancer screening and found it effective in people with lower education levels, too. Trevena et al. found no evidence of any interaction when adequate knowledge was significantly improved by the decision aid across all groups with different educational statuses. Kellar and Mason [12] stated that even an informed choice invitation for Type 2 diabetes screening, developed for ease of readability, may be a disadvantage for individuals without higher education. Evaluation of this invitation was carried out in comparison with a standard invitation. Regarding attendance at screening for diabetes, the interaction between type of invitation and social deprivation was not significant. Attendance for screening and intentions to engage in lifestyle change was inversely associated with deprivation [13, 14].It has to be considered that our study was not designed to detect interactions of the socioeconomic status with the intervention, randomization or case number. Hence, maybe interactions could not be detected due to less power, which is underlined by the large confidence intervals in Figure 1. Further limitations have to be considered. Income and occupation could not be analyzed. A selection bias cannot be excluded, as the association analyses were carried out on the subpopulation (n = 647) with complete values of all knowledge items and no missing values on the potential confounders. The included participants were significantly younger compared to the excluded participants, and had better knowledge before intervention (p = 0.025), were more frequently vocationally trained and employed, but had no significant differences in education. As visitors to health-related websites and also members of the “Techniker Krankenkasse” may belong to higher social classes, they cannot be taken as representative for the entire population. Hence, a bias due to the recruitment itself has to be considered, which could affect the whole study population but not the difference between intervention and control groups. However, different potential confounders without missing values were recognized in the multiple regression analysis, so that the results are bias-adjusted for these variables, also with respect to dropout.

In conclusion, the effect of an EBPI about primary prevention of diabetes on knowledge as the main parameter for an informed decision was not significantly affected by socioeconomic variables. However, there was a tendency to reduced intervention effects in low educated individuals. Possibly the power was too low to detect interaction effects. Larger studies using SES-specific designs are needed to clarify the effect of SES. We suggest considering the socioeconomic status when evaluating a decision aid, e.g. an EBPI, to ensure its effectiveness not only in higher socioeconomic groups and to find out whom the promotion of decision aids, especially EBPI, may benefit or harm.
